# Tumor associated neutrophils promote prostate cancer progression by mediating neutrophil trap secretion through PSMA1- NF-κB-HIF-1α signaling axis

**DOI:** 10.3389/fimmu.2025.1467357

**Published:** 2025-08-18

**Authors:** Qian Dai, Hua Wang, Fang Li, Runchun Huang, Chenjun Jiang, Liuya Yuan, Yayun Wang, Xun Li

**Affiliations:** ^1^ The First Clinical Medical College, Lanzhou University, Lanzhou, China; ^2^ Department of Urology, First Hospital of Lanzhou University, Lanzhou, China; ^3^ Department of General Surgery, The First Hospital of Lanzhou University, Lanzhou, China

**Keywords:** prostate cancer, neutrophil extracellular trap, prognostic signature, neutrophil, tumor organoid

## Abstract

Prostate cancer (PCa) is a common and deadly cancer in men, and despite its low specificity, PSA testing is the main method that is used to predict prognosis. Effective methods for predicting prognosis in clinical practice are lacking. Here, ① in this retrospective analysis of clinical data of PCa patients, we discovered that patients with PCa have elevated neutrophil levels and a greater risk of complications than patients with prostatic hyperplasia. ② We integrated LASSO regression analysis and machine learning analyses to create a prognostic prediction model involving 6 genes, and this model effectively categorized patients into high-risk and low-risk groups, with higher risk scores indicating a poorer prognosis. Furthermore, we used multivariate regression analysis to confirm that the risk score was an independent prognostic factor and created nomograms on the basis of clinical characteristics. Notably, the deconvolution algorithm revealed different compositions of the tumor microenvironment, with a greater proportion of neutrophils observed in the high-risk group. ③ Finally, we conducted single-cell sequencing analysis and established a prostate cancer organoid model to confirm that TANs may exacerbate the TME in PCa via neutrophil trap formation, which is mediated by the PSMA1-NF-κB-HIF-1α signaling axis. Overall, this novel NET-related signature of PCa provides new insights for in-depth understanding and prediction of PCa prognosis.

## 1 Introduction

Prostate cancer (PCa) is the most common cancer among men worldwide; PCa accounts for 29% of all cancer cases, and its mortality rate is 11%, second only to that of lung cancer, which accounts for 21% of all cancer-related deaths (1). In 2020, the number of newly diagnosed PCa cases worldwide was approximately 1.414 million, and approximately 375000 PCa-related deaths were recorded (2). For patients with PCa in the early stages, there are multiple treatment options, such as chemotherapy, radiation therapy, prostatectomy, and androgen deprivation therapy (3). However, 30%50% of patients experience biochemical recurrence (BCR) after treatment, and most patients with advanced PCa are resistant to androgen deprivation therapy and chemotherapy (4). Early diagnosis or detection of clinical progress when the disease burden is relatively low and when the treatment effect is optimal has enormous clinical value. Serum levels of prostate specific-antigen (PSA) have long been used as a biomarker for screening and diagnosing PCa, but the poor specificity of this biomarker has led to unnecessary biopsy and severe overtreatment of low-risk PCa (5). Currently, effective prognostic prediction methods for use in clinical practice are lacking. Therefore, an effective PCa diagnostic and prognostic biomarker for identifying high-risk PCa in early stages and implementing appropriate treatment methods is crucial for prolonging survival and reducing mortality among PCa patients.

Neutrophils, which are the most abundant type of endogenous immune effector cell, respond to various pathogens. Neutrophils release a structure called neutrophil extracellular traps (NETs) through a type of regulated cell death called “NETosis” (6). NETs are extracellular network structures that contain DNA chromatin complexes and are extruded from dying neutrophils. NETs were originally described as mechanisms for capturing, limiting, and killing invading bacteria and other pathogens (7, 8), but with increasing research, NETs have been shown to play controversial roles in cancer, including protecting cancer cells in certain situations, reducing the effectiveness of immunotherapy, and participating in the progression and metastasis of cancer (9–11). Many studies have predicted the prognosis of breast cancer, lung adenocarcinoma, and gastric cancer by scoring NET-related gene datasets, but the value of NETs in predicting PCa prognosis remains unclear (12–14).

In this study, we aimed to identify prognostic biomarkers associated with NETs and construct NET risk models to analyze tumor-related prognosis, TME composition, and drug sensitivity in PCa patients. Furthermore, we confirmed that the risk score was an independent prognostic factor and created nomograms on the basis of clinical characteristics. We also utilized single-cell data to analyze the potential subcluster localization and biological functions of the NET-DEGs in the TME of PCa, revealing potential strategies for personalized diagnosis and treatment in clinical practice.

## 2 Materials and methods

### 2.1 Data acquisition and preprocessing

The RNA sequencing (RNA-seq) data of 502 PCa samples and 100 normal Prostate samples with their clinicopathological parameters were downloaded from the cancer genome atlas (TCGA, https://portal.gdc.cancer.gov) and Genotype-Tissue Expression (GTEx, https://www.gtexportal.org/home/) databases.

Single-cell RNA sequencing data were obtained from the GSE181294 (https://www.ncbi.nlm.gov/geo/query/acc.cgi?acc=GSE181294) dataset, which comprised a total of 38 samples. Initially, samples from different sequencing batches were excluded, and one sample without annotated cell type information was removed. Ultimately, 14 normal samples and 18 tumor samples were retained for subsequent analysis.

### 2.2 Identification of differentially expressed neutrophil extracellular traps related genes

The “DESeq2” package was used to identify differentially expressed genes (DEGs) in 502 PCa samples and 100 normal Prostate samples. P < 0.05 and |log_2_ fold change (FC)|> 1 were set as cut-off values. On the other hand, crossover between neutrophil extracellular traps related genes and DEGs was performed to obtain neutrophil extracellular traps related genes DEGs (NET-DEGs); the latter neutrophil extracellular traps related genes (n = 438) were extracted from Kyoto Encyclopedia of Genes and Genomes(KEGG), Gene Ontology(GO), GSEAMSigDB, paper and GeneCard.

### 2.3 Enrichment analysis of differentially expressed neutrophil extracellular traps related genes

The biological process enrichment of 84 NET-DEGs were analyzed with Gene Ontology (GO) and Kyoto Encyclopedia of Genes and Genomes (KEGG) through R statistical software including “clusterProfiler”, “org.Hs.eg.db”, “enrichplot”, “ggplot2”, and “GOplot” packages.

### 2.4 Identification of prognostic genes

To investigate the relationship between the expression levels of neutrophil extracellular traps related genes and biochemical recurrence of PCa patients, we conducted a univariate Cox regression analysis using the “survival” package. A significant filtering criterion was set at p < 0.01 for further analysis. To ensure the robustness of this selection, we adopted a bootstrap approach by sampling 80% of patients 1000 times and only retrieved genes with P < 0.01 more than 800 times.

### 2.5 Survival analysis of prognostic genes

For Kaplan–Meier curves, *P*-values and hazard ratios (HRs) with 95% confidence intervals (CIs) were calculated *via* the log-rank test. HRs of > 1 indicated risk factors, whereas HRs of < 1 indicated protective factors. The R packages ‘survival’, ‘survminer’ and ‘timeROC’ were used for survival analysis. *P*-values of < 0.05 were considered statistically significant. More importantly, 1-year, 3-year and 5-year prognostic values for biochemical recurrence, survival-dependent subject operating characteristic (ROC) curves and calibration curves were used to evaluate the prognostic genes in the TCGA training set.

### 2.6 Machine learning to build prognostic signatures

Six machine learning algorithms—Elastic Net (Enet), Lasso, Ridge regression, CoxBoost, partial least squares Cox regression (plsRcox), and supervised principal components (SuperPC)—were employed to develop prognostic models for biochemical recurrence using a cohort of 486 prostate cancer patients from The Cancer Genome Atlas (TCGA; https://portal.gdc.cancer.gov). The cohort was randomly partitioned into a training set (70%, n=340) and an independent validation set (30%, n=146). Each algorithm underwent model fitting using the training set with hyperparameter optimization conducted via 10-fold cross-validation, where a grid of candidate values was evaluated by calculating Harrell’s Concordance Index (C-index) on held-out folds to identify the parameter combination that maximized mean cross-validated C-index. Key hyperparameters tuned included penalty strength for all algorithms, L1/L2 mixing parameters for Enet, boosting steps for CoxBoost, principal components for plsRcox and SuperPC, and feature selection thresholds for SuperPC. Following hyperparameter selection, final models were retrained using the full training set and their prognostic performance was assessed on the validation set. Through comparative evaluation of validation set C-index values, Lasso-Cox regression was selected as the optimal approach. The Lasso-Cox model development involved refitting the model to the entire training set with penalty parameter optimization via 10-fold cross-validation minimizing partial likelihood deviance, followed by gene selection based on non-zero coefficients to establish the final signature. Risk scores were computed using the linear predictor formula η = β1 × Gene1 Expression +… + βn × Genen Expression, and patients were stratified into high-risk and low-risk groups using the training set’s median risk score as the threshold. All packages came from this website (https://cran.r-project.org/web/packages/index.html).

### 2.7 Construction of nomogram and calibration curves

Use the “RMS” package of R software to construct a column chart to predict individual survival probability, and draw calibration curves for predicting 1-year, 3-year, and 5-year survival rates of PCa patients.

### 2.8 Immunological correlation analysis

First of all, we calculated the immune score, matrix score, estimated score, and tumor purity using the ESTIMATE algorithm. Subsequently, We used MCPCOUNT and EPIC to evaluate the differences in immune cell components in the PCa microenvironment (PCa TME) between high and low risk groups.

### 2.9 Drug sensitivity analysis

Based on transcriptome data from PCa samples, drug sensitivity was analyzed using the R package “oncoPredict”, Genomics of Drug Sensitivity in Cancer (GDSC) database, and The Cancer Therapeutics Response Portal (CTRP) database.

### 2.10 scRNA-seq data analysis and cell types identification

A series of quality control steps were performed to filter out cells that did not meet the following criteria: a gene expression count exceeding 500, a Unique Molecular Identifier (UMI) count exceeding 500, or mitochondrial gene expression exceeding 10. After importing the data matrix into R, we conducted data normalization using Seurat software version 4.4.0. We selected the top 2000 highly variable genes for scaling and, considering batch effects among samples, applied the “harmony” package for batch correction. Following normalization, we performed dimensionality reduction using the top 20 principal components (PCs) and subsequently carried out density-based clustering to identify cell groups. An appropriate resolution parameter was chosen for the initial clustering. Different cell types were defined based on specific marker genes as follows: In accordance with a set of appropriate marker genes, we defined distinct cell types as follows: “MSMB,” “KLK3,” and “KLK4” were employed to define Epithelial Cells; “KRT19,” “KRT15,” and “ELF3” were used to define Basal Epithelial Cells; “CD3D,” “CD2,” “CCL5,” and others were utilized to define T_NK Cells. Furthermore, we also defined Macrophages, Mast Cells, Endothelial Cells, Neutrophils, B Cells, Plasma Cells, Fibroblasts, and Smooth Muscle Cells. Next, we calculated the Ro/e values of each neutrophil subgroup to evaluate their distribution trends in tumor and normal tissues. Meanwhile, The Monocle2 algorithm is used to calculate the differentiation direction between various neutrophil subpopulations.

### 2.11 Module score calculation

We used the Seurat package’s AddModuleScore function to compute pathway-related gene module scores in single-cell samples, indicating their activity. We compared these scores between tumor-associated and normal neutrophil subtypes and assessed significance with Wilcox method.

### 2.12 Cell culture

HL-60 (RRID: CVCL_0002), 22RV1 (RRID: CVCL_1045) and LNCaP (RRID: CVCL_0395) cell lines were obtained from the Cell Bank of Shanghai Institute of Biotechnology, Chinese Academy of Sciences (Shanghai, China). Neutrophils were autonomously harvested from the blood of healthy donor through a process involving CD66b Biotin antibody (Biolegend, 305120) staining, the addition of biotin magnetic beads, and sorting using MS columns (Miltenyi Biotec, 130-042-201). HL-60, 22RV1, LNCaP and neutrophils cells were cultured in RPMI-1640 medium with 10% FBS(Gibco) and antibiotics (Beyotime, penicillin, 100U/ml; streptomycin, 0.1mg/ml). In co-culture experiments, we used 24 well and 6 well transwell plates with 0.4 µm pore polyester membrane insert (Corning), in which HL-60 cells and neutrophils were cultured in upper chambers, and PCa cell lines were cultured in lower chambers.

### 2.13 Knockdown of PSMA1 in HL-60 cells

The ORFs of human genes PSMA1 was seamlessly cloned into pLV3-CMV-3×FLAG-CopGFP Puro using cloning technology. Knockdown plasmid, co-transfection of psPAX2 and pMD2.G into 293T cells for packaging lentivirus. After 48 hours, collect the supernatant virus solution and concentrate the virus. After infecting HL-60 virus with concentrated virus solution for 24–48 hours, green fluorescence was observed in the HL-60 cell line. Stable knockout cell lines were obtained by screening with 1ug/mL puromycin, and the knockout efficiency was verified by qPCR and WB.

### 2.14 Real-time RT-PCR analysis

Total RNA was extracted with TRIzol (Invitrogen). 1 mg RNA was used as template for cDNA conversion with HiScript III RT SuperMix (+gDNA wiper) (Vazyme). Real-time RT-PCR analysis of mRNA expression was performed in triplicate with AceQ Universal SYBR qPCR Master Mix (Vazyme). Gene expression was normalized to β-actin as endogenous control.

### 2.15 Western blotting

Total protein was extracted using RIPA buffer (epizyme, China) with protease inhibitor cocktail (Roche Applied Science, Switzerland), and quantitated by bicinchoninic acid (BCA) protein assay (epizyme, China).

### 2.16 Cell proliferation assay

For colony formation assay, 22RV1 and LNCaP cells were seeded in 6-well plates (5000 cells/well), and control and PSMA1 knockdown HL-60 cells were cultured in upper chambers in transwell plates. After 1–2 weeks of co-culture, colonies were stained with crystal violet (Beyotime, C0121).

### 2.17 Migration and invasion assay

After co-culturing with control and PSMA1 knockdown HL-60 cells for 3 days, 22RV1 and LNCaP cells were loaded in the upper chamber of transwell plates or the wells of a Matrigel plate (8μm pore size; Corning), and incubated at 37°C for 36~48h. Migrated cells were stained with crystal violet, and counted in 3 random high power fields.

### 2.18 Organoid culture, coculture

PCa tumor tissue was collected, washed twice with PBS, and then minced in a Petri dish with shears into fragments of 1–3 mm3 or smaller. The fragments were transferred to a 15 mL Falcon tube with 5 mL of tumor tissue digestion solution. Then, tissue fragments were incubated in the solution at 37°C for 15–20 minutes and intermittently mixed every 5 minutes. Digestion was evaluated with light microscopy for dissociated fragments. Once at least 80% single cells were present in the mixture, PBS was added and filtered using a 100 μm cell strainer. The supernatant was transferred to a separate 15 mL Falcon tube and centrifuged at 300 x g for 5 minutes at 4°C. Next, the pellet was resuspended evenly in a 70:30 mixture of Matrigel^®^ Matrix (Corning, Catalog 356231). Organoid media were placed on ice to prevent polymerization of the mixture prior to plating. The mixture was plated into 50 µL domes on a prewarmed 24-well plate (Corning, Catalog 3524). The plate was placed in a CO2 incubator (37°C, 5% CO2) for 10 minutes to allow the Matrigel to polymerize and solidify, after which 0.75 mL of PCa organoid culture medium (JFKR, Catalog JFKR-PCA-100, Shanghai, China) was added to tumor PCa organoid wells. The culture medium was changed every 2–3 days, and after 10 days of culture, the organoids were harvested for further processing.

Human PCa organoid culture medium (JFKR, Catalog FKR-PCA-100) contained Advanced DMEM-F12 (Gibco, cat. 12634-028), 50 ng/L Wnt3A (PeproTech, 315-20-2UG), 1% Glutamax (Life Technologies, Catalog 35050061), 1% HEPES ((Gibco, cat. 15630-056), 1% penicillin–streptomycin (Gibco, cat. 15070063), 2% B27 (Gibco, cat. 17504-044), 1% N2 (Gibco, cat. 17502048), 1% insulin-transferrin-selenium (Gibco, cat. 41400045), 0.2% Primocin (Gibco, cat. PML-40-60), 50 ng/mL human EGF (Peprotech, cat. AF-100-15), 100 ng/mL human FGF10 (Peprotech, cat. 100-26), 1.25 mM N-acetyl-L-cysteine (Sigma–Aldrich, cat. A9165-5G), 1 mM nicotinamide (Sigma–Aldrich, cat. N0636), and 0.5 mM A83-01 (Tocris, cat. 2939).

PCa organoids were harvested, mechanically broken and transferred into each well of a 24-well plate containing 250 μl of HL-60 medium. HL-60 cells were harvested, counted and combined with organoids at a 25-50:1 (HL-60 cells:organoids). Coculture with HL-60 cells was performed for 1–3 d at 37°C and 5% CO2.

### 2.19 Statistical analysis

The association between two variables was assessed by Pearson correlation analysis. Kaplan-Meier survival curves and log-rank tests were used to compare overall survival in different patient groups. Linear models were generated for comparisons between two groups were performed with Student’s t test (for normally distributed data) or Mann-Whitney U test (for non-normally distributed data). Benjamini & Hochberg correction was performed for multiple comparison.

## 3 Results

### 3.1 Clinical cohort: PCa patients exhibited a greater proportion of neutrophils than controls

In order to study the clinical characteristics of prostate cancer, we conducted a retrospective analysis of 307 prostate cancer patients from the Department of Urology at the First Hospital of Lanzhou University from 2013.03 to 2024.12 and 214 prostate hyperplasia patients from 2019.04 to 2023.10 (**Table 1**). The results revealed that the level of neutrophils in PCa patients was greater than that in prostatic hyperplasia patients (4.25*1*10^9^ *vs*. 3.84*1*10^9^, P<0.05), and the proportion of neutrophils among white blood cells was also greater (67.77% *vs*. 63.60%, P<0.01). Furthermore, on the basis of the 25% neutrophil count, PCa patients were divided into a high-neutrophil-count group and a low-neutrophil-count group. Interestingly, we found that PCa patients with high neutrophil counts had higher Gleason scores and greater incidence of pain, difficulty urinating, and all three (pain, difficulty urinating and fatigue) than those with low neutrophil counts, suggesting a close correlation between higher neutrophil counts and poorer clinical manifestations in PCa patients.

**Table 1:** Analysis of clinical data of prostate cancer patients.

Comparison items	Subgroup		Statistical differences	
Laboratory examination	Prostate cancer (n=307)	Prostate hyperplasia (n=214)	X^2^/t	P value
White blood cell count (1*10^9^)	6.07 ± 1.88	5.93 ± 1.97	0.815	<0.001
Neutrophil percentage	67.77% ± 11.12%	63.60% ± 10.24%	4.350	<0.001
Neutrophil count (1*10^9^)	4.25 ± 1.81	3.84 ± 1.69	2.569	0.010
Clinical features of prostate cancer	High neutrophil count (n=77)	Low neutrophil count (n=230)	X^2^/t	P value
Complications (pain)	36 (46.75%)	68 (29.57%)	7.608	0.006
Complications (fatigue)	36 (46.75%)	100 (43.49%)	0.251	0.617
Complications (difficulty urinating)	67 (87.01%)	174 (75.65%)	4.412	0.036
Complications (all three present)	35 (45.45%)	68 (29.57%)	6.533	<0.001
Gleason score	8.31 ± 1.13	7.57 ± 1.10	5.084	<0.001
Lymph node metastasis	26 (33.77%)	79 (34.35%)	0.009	0.926
Bone metastasis	35 (45.45%)	98 (42.61%)	0.19	0.663

### 3.2 Identification and functional enrichment analysis of NET-DEGs in PCa

**Figure 1:**
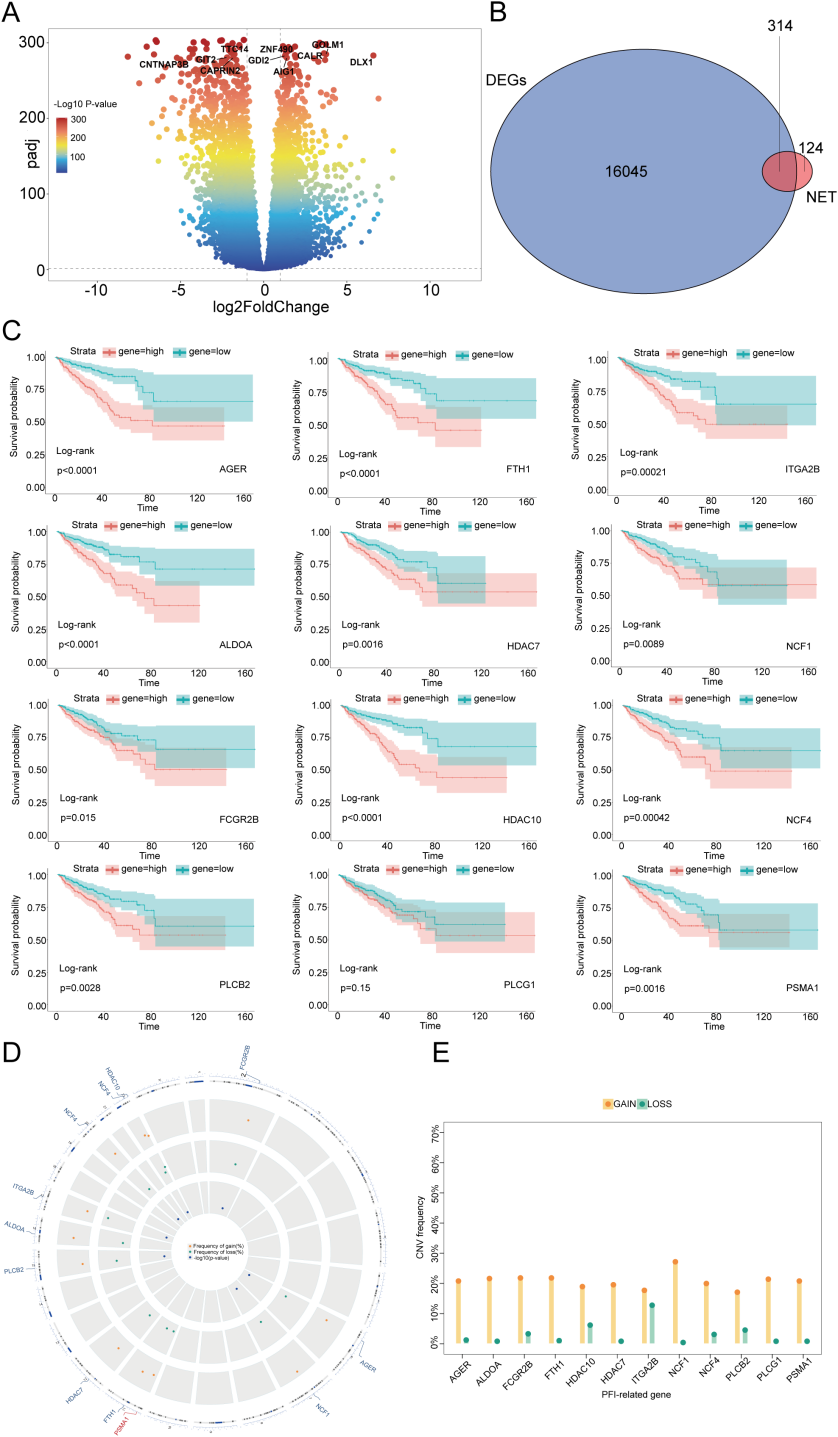
Identification of NET-DEGs in PCa and prognostic characteristics of these genes. **(A)** Volcano plot of DEGs in PCa: the horizontal axis represents the logarithmic fold change (log2FoldChange), and the vertical axis represents the negative logarithm of the P value (-log10 P value). Each point represents a gene, and the colors represent different ranges of P values. Several genes of interest are marked at the top. **(B)** Venn diagram of the intersection of DEGs and NET-related genes in PCa. The overlapping number (314) between the two represents genes that belong to both DEGs and NETs. **(C)** Kaplan–Meier plots demonstrating the impact of different gene expression levels on the survival rate. Each subgraph represents a gene, and the red and blue lines represent the survival probabilities of groups with high and low expression, respectively. The P value of the log rank test is also indicated in the graph for statistical significance testing. **(D)** CNV locations on the 23 chromosomes of the 12 NET-DEGs. **(E)** Frequencies of CNV gain and loss among 12 NET-DEGs. Yellow indicates an increase in gene copy number (GAIN), and green indicates a decrease in gene copy number (LOSS).

Considering these clinical features, we were inspired to further explore neutrophils and NETs. To reveal the characteristics of NETs in PCa, we performed differential expression analysis on a dataset comprising 502 cancerous and 100 normal prostate samples, and we identified 16,045 DEGs (**Figure 1A**). We intersected the collected NET-related genes (7, 15) with 16045 DEGs and successfully identified 314 NET-DEGs (**Figure 1B**). To elucidate the biological functions of these NET-DEGs, we conducted GO and KEGG pathway enrichment analyses. GO analysis revealed that the NET-DEGs were predominantly involved in processes related to the regulation of the inflammatory response and the response to external stimuli, suggesting that NET-DEGs play a pivotal role in orchestrating human immune system responses (**Supplementary Figure S1A**). In addition, KEGG pathway analysis revealed the involvement of NET-DEGs in neutrophil extracellular trap formation and Fc gamma R-mediated phagocytosis (**Supplementary Figure S1B**), further indicating the role of NET-DEGs in the immune response to PCa.

### 3.3 Identification and survival analysis of prognostic NET-related PCa DEGs

To investigate the correlation between NET-DEGs and PCa prognosis, we conducted univariate Cox regression and used the Boruta algorithm to identify 12 NET-related prognostic genes (NET-RPGs): AGER, ALDOA, FCGR2B, FTH1, HDAC7, HDAC10, ITGA2B, NCF1, NCF4, PLCB2, PLCG1, and PSMA1. Copy number variation (CNV) analysis revealed that the scores of these 12 genes were increased (**Figure 1E**), suggesting that these genes may serve as potential risk genes and were associated with poor PCa prognosis. Furthermore, the spatial distribution of these CNVs and their genomic positions are shown in **Figure 1D**, highlighting the extensive nature of these genomic alterations and structural variations at the submicroscopic level of chromosomes. To further elucidate the relationship between the 12 NET-DEGs and patient prognosis, we conducted Kaplan–Meier survival analysis and found that high expression of the 12 genes was associated with poor prognosis. This result highlighted that these 12 genes are vital factors that affect prognosis and have potential to be foundational elements for developing prognostic prediction models (**Figure 1C**, **Supplementary Figure S2**). To further validate the expression of the 12 genes in clinical samples, PCR analysis was performed; the results revealed that AGER, ALDOA, FCGR2B, FTH1, HDAC7, HDAC10, ITGA2B, NCF1, NCF4, PLCB2, and PLCG1 were downregulated and that PSMA1 was upregulated in PCa tissues, which was consistent with the results of the bioinformatics analysis (**Supplementary Figure S3**).

### 3.4 Construction of a prognostic model based on NET-DEGs in the training set

**Figure 2:**
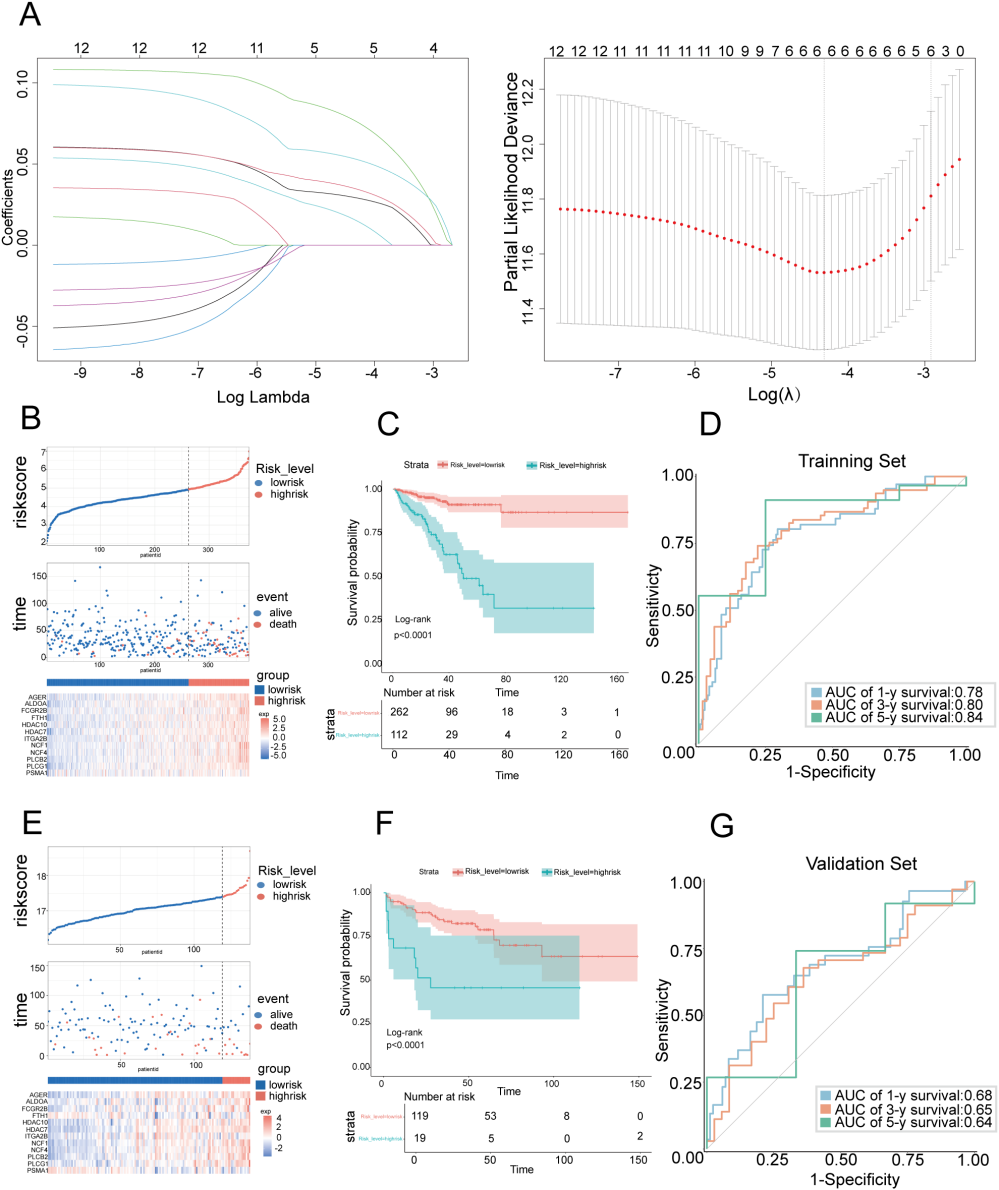
Construction and validation of prognostic features for NET-DEGs **(A)** LASSO regression analysis of the prognosis-related genes. The left subgraph shows the variation in feature coefficients with log lambda (regularization parameter) in the LASSO regression model (6 genes in the risk score model: PLCG1, PLCB2, AGER, ALDOA, FCGR2B, and PSMA1). The subgraph on the right is a cross-validation graph that displays the partial likelihood deviation for different lambda values. **(B)** Risk score distribution chart for the TCGA PCa cohort. The top figure shows the risk score for each sample (red indicates high risk, and blue indicates low risk). The middle graph shows the distribution of survival status (alive and dead) over time. The bottom figure is a heatmap that shows the changes in gene expression levels in high-risk populations. **(C)** Kaplan–Meier survival curve showing the variation in survival probabilities over time for high-risk and low-risk patients in the TCGA PCa cohort. **(D)** ROC curve used to evaluate the predictive performance of the model on the training set. The area under the curve (AUC) demonstrates the accuracy of the model in predicting 1-year, 3-year, and 5-year survival rates. The AUC values were 0.78, 0.80, and 0.84, respectively, in the TCGA PCa cohort. **(E)** Risk score distribution chart for the GEO PCa cohort. The top figure shows the risk score for each sample (red indicates high risk, and blue indicates low risk). The middle figure shows the distribution of survival status over time. The bottom figure is a heatmap that shows the changes in gene expression in high-risk populations. **(F)** Kaplan–Meier survival curve showing the variation in survival probabilities over time for high-risk and low-risk populations in the GEO PCa cohort. **(G)** ROC curve used to evaluate the predictive performance of the model on the training set. The area under the curve (AUC) demonstrates the accuracy of the model in predicting 1-year, 3-year, and 5-year survival rates. The AUC values were 0.68, 0.65, and 0.64, respectively, in the GEO PCa cohort.

To integrate the comprehensive impact of the 12 genes on prognosis, we constructed a risk prediction model involving 6 NET-DEGs using LASSO Cox regression (**Figure 2A**). Using TCGA-PARD data as the training set, we divided PCa patients into a high-risk group (n=112) and a low-risk group (n=262) on the basis of the risk model (**Figure 2B**). Kaplan–Meier survival analysis further confirmed that the high-risk patients had a worse prognosis than their low-risk counterparts (**Figure 2C**). The predictive ability of the model was underscored by time-dependent ROC analysis, which revealed AUC values of 0.78 at 1 year, 0.80 at 3 years, and 0.84 at 5 years (**Figure 2D**). These findings confirmed the informative nature of NET-DEG signatures and validated the accuracy of the model in predicting PCa prognosis within the training set.

### 3.5 Validation of predictive models in the test set

To assess the reliability of our prognostic model, we evaluated the model on an independent test set comprising 150 PCa patients sourced from the GSE210343 dataset. Using the same risk score formula derived from the training set, we stratified these patients into a high-risk group and a low-risk group, which was consistent with the training set, suggesting a correlation between the gene expression level and patient risk stratification (**Figure 2E**). Kaplan–Meier survival analysis revealed that the high-risk group had a poorer prognosis than the low-risk group, which was consistent with the training set results (**Figure 2F**). Time-dependent ROC analysis was employed to quantify the accuracy of the model in predicting biochemical recurrence across different time frames (**Figure 2G**). The analysis yielded AUC values of 0.68 at 1 year, 0.65 at 3 years, and 0.64 at 5 years, demonstrating that the model maintained an acceptable level of predictive accuracy over time, even in an independent test set. These results highlighted the validity and applicability of our prognostic model, confirming its potential for use as a reliable tool for predicting PCa prognosis in clinical settings.

### 3.6 Construction of a predictive nomogram model

**Figure 3:**
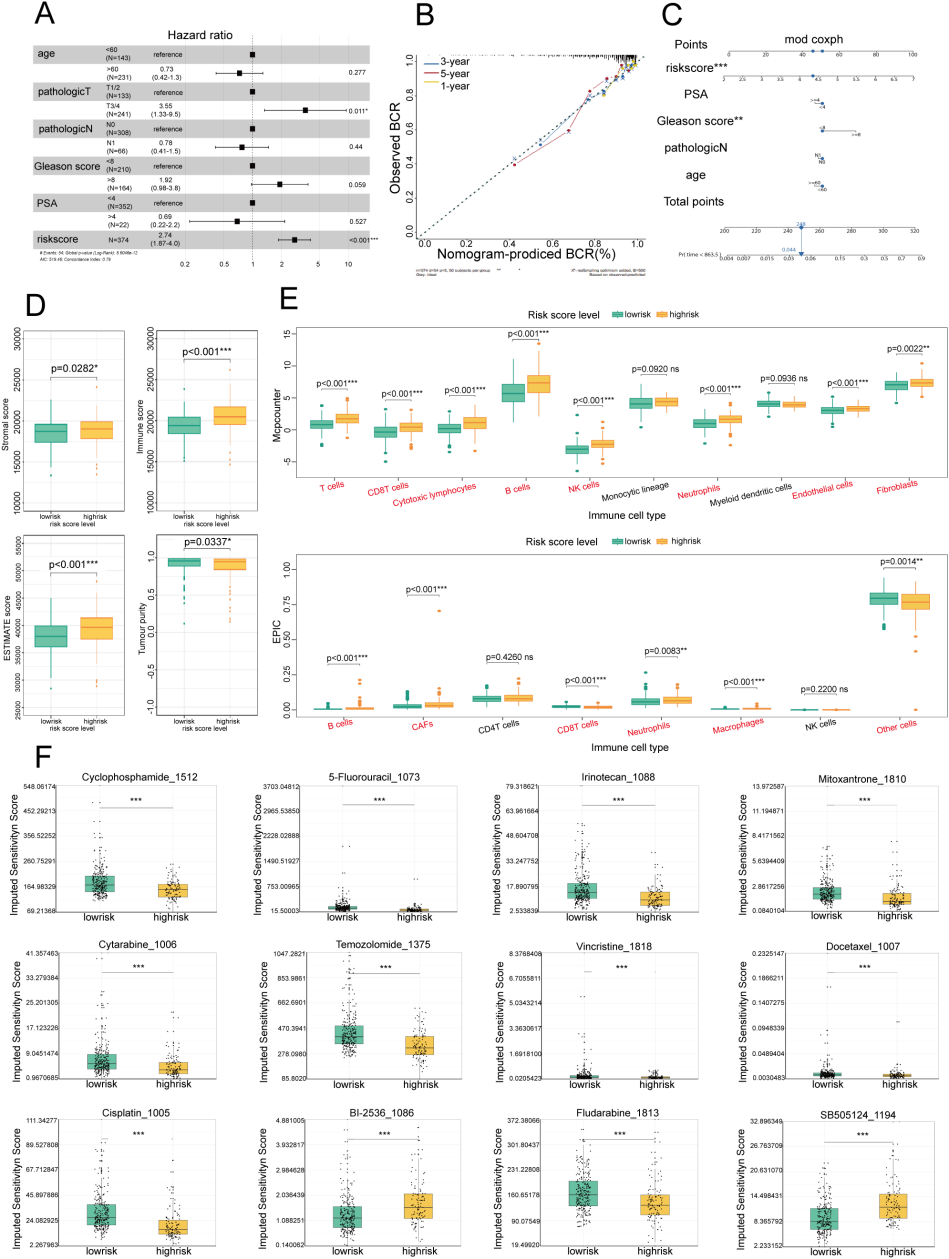
Establishment of a prognostic nomogram for the survival of patients with PCa and analysis of immune cell infiltration and drug sensitivity in high- and low-risk groups **(A)** Univariate Cox analysis of the risk score model in the TCGA and GEO cohorts. **(B)** Calibration curve for evaluating the accuracy of the nomogram model. The horizontal axis represents the biochemical recurrence (BCR) probability predicted by the model, and the vertical axis represents the actual observed BCR probability. **(C)** Nomogram for predicting the BCR of PCa: Each variable, such as the risk score, PSA level, and Gleason score, corresponds to a certain number of points, and the total points can be converted to the corresponding survival probability on the total points axis below the graph. **(D)** Box plot showing the distributions of overall survival, immune scores, and stromal scores among patients with different risk levels (low- and high-risk). Green represents the low-risk group, and yellow represents the high-risk group. **(E)** Box plot showing the distribution of different immune cell types in low-risk and high-risk patients. Green represents the low-risk group, and yellow represents the high-risk group. **(F)** Box plot showing the sensitivity scores of different drugs in low-risk and high-risk patients. Green represents the low-risk group, and yellow represents the high-risk group. *P<0.05; **P<0.01; ***P<0.001.

To determine the standalone prognostic capability of risk scores for predicting biochemical recurrence (BCR) in PCa patients, we performed univariate Cox regression analyses across both the training and test sets. BCR in PCa refers to the situation where there is evidence of cancer returning after initial treatment, based solely on increasing levels of prostate-specific antigen (PSA) in the blood, without any physical or radiological signs of cancer (16). The analyses demonstrated a profound association between the risk score and the time to biochemical recurrence in both sets, and the risk score served as an independent prognostic indicator (**Figure 3A**). To increase the clinical utility of the model and offer a more quantitative approach for predicting PCa patient outcomes, we developed a comprehensive nomogram. This graphical representation integrated the risk score with other critical clinical parameters, including PSA levels, Gleason score, age, and lymph node involvement (N) (**Figure 3C**). The nomogram highlighted the Gleason score and risk score as particularly influential factors in determining prognosis. Furthermore, the predictive accuracy of the nomogram was validated through the construction of calibration curves. These curves compared the 1-year, 3-year, and 5-year prognosis predictions of the nomogram against actual patient outcomes, and they demonstrated a high degree of concordance (**Figure 3B**). The calibration curves confirmed the reliability of our risk score-based model in accurately predicting patient prognosis. The integration of risk scores into a user-friendly nomogram offered substantial advancements in the personalized prediction of biochemical recurrence times for PCa patients, which shows promise for enhancing decision-making processes in clinical settings.

### 3.7 Comparison of immune cell infiltration into the microenvironment in the high-risk and low-risk groups

#### 3.7.1 High-risk patients have a poor immune status

To further examine the biological characteristics of PCa patients and assess the immune efficacy of the high- and low-risk groups, an immune infiltration analysis was conducted. Using the ESTIMATE algorithm, we compared four key immune status indicators—immune score, stromal score, estimate score, and tumor purity—between the two groups (**Figure 3D**). Our findings revealed that the high-risk group exhibited considerably higher immune and ESTIMATE scores, indicating that the high-risk group had increased immune and stromal cell infiltration. This pattern suggested that patients in the high-risk category may benefit more from immunotherapeutic approaches, given the richer immune and stromal cell milieu within the TME.

#### 3.7.2 Differences in tumor infiltration and immune cells between the high- and low-risk groups

To further elucidate the cellular composition of the TME in the high-risk and low-risk groups, we employed MCP-counter and EPIC analyses (**Figure 3E**). The results revealed that immune cells were more predominant in the high-risk group than in the low-risk group, suggesting a more complex and dynamic immune landscape. Furthermore, the elevated neutrophil infiltration observed in the high-risk group through the MCP-counter and EPIC analyses was consistent with our emphasis on NETs, highlighting the importance of neutrophils and their associated extracellular traps in shaping the immunological landscape of PCa.

### 3.8 Sensitivity analysis of chemotherapeutic drugs

Given the established correlation between varying levels of immune cell infiltration and chemotherapy sensitivity in PCa (17), tailoring chemotherapy treatments to specific patient populations on the basis of their risk profile has become imperative. Our investigation into the sensitivity of high- and low-risk patients to common chemotherapeutic agents revealed a marked difference in drug responsiveness, highlighting the potential for personalized treatment approaches. In the high-risk group, there was pronounced sensitivity to a broad spectrum of commonly used chemotherapeutic drugs, including cyclophosphamide, 5-fluorouracil, cisplatin, vincristine, cytarabine, temozolomide, irinotecan, fludarabine, and docetaxel (**Figure 3F**), suggesting that these agents may be particularly effective for treating patients who are identified as high risk, potentially improving therapeutic outcomes in this set. Conversely, the low-risk group exhibited greater sensitivity to BI-2536 and SB-505124 (**Figure 3F**). This distinction in drug responsiveness emphasized the necessity of adopting a risk-based approach to chemotherapy, ensuring that patients receive the most effective treatment for their specific disease profile. Our comprehensive analysis included a total of 156 chemotherapeutic drugs, revealing significant differences in treatment sensitivity between the high- and low-risk groups. These findings reinforce the concept that risk scores can be a valuable tool for guiding the selection of chemotherapeutic agents, enabling the customization of treatment plans to optimize patient outcomes in PCa.

### 3.9 Single-cell sequencing of the cellular composition of the PCa TIME

**Figure 4:**
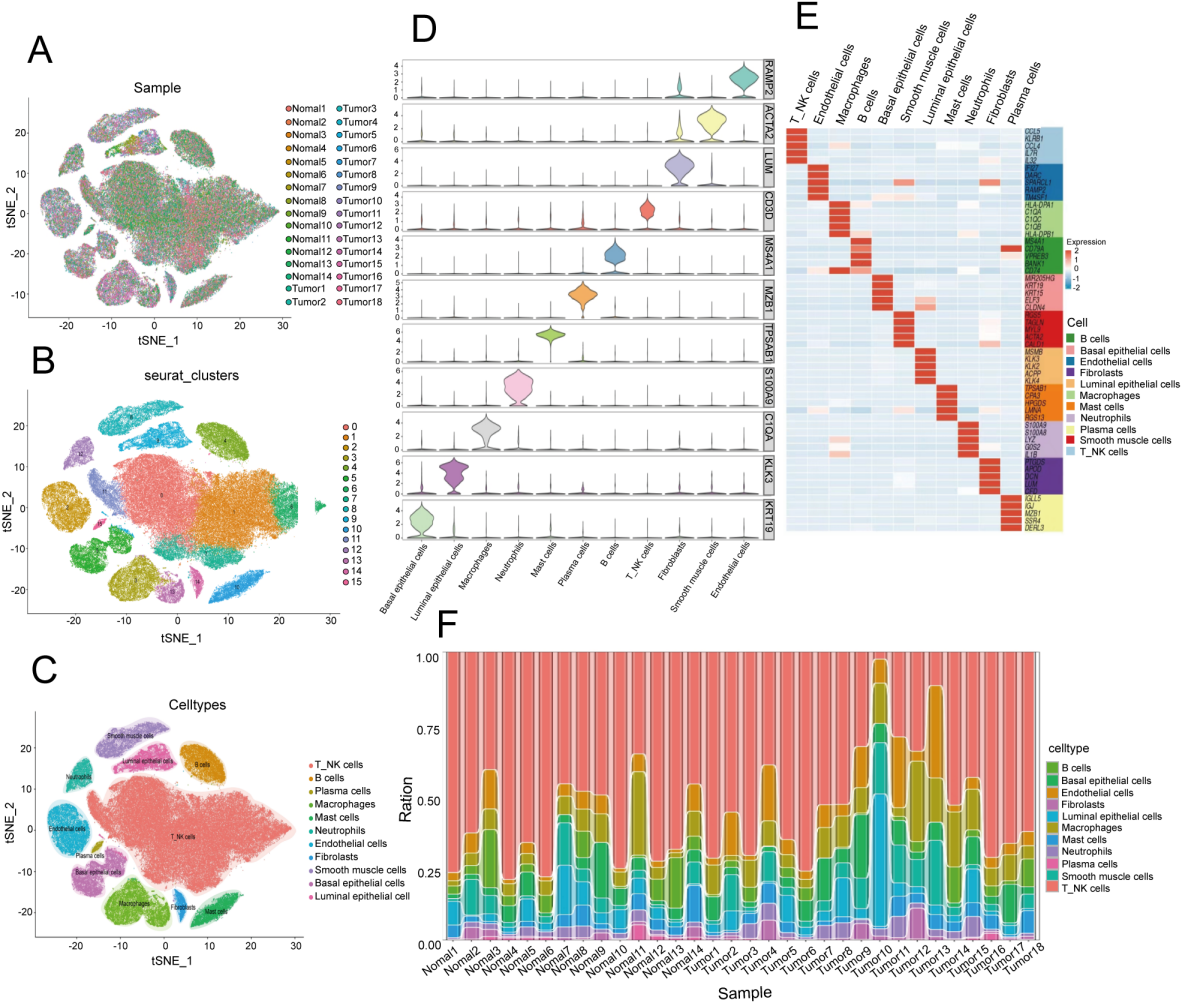
Single-cell transcriptome landscape in the prostate cancer microenvironment. **(A)** t-distributed stochastic neighbor embedding (t-SNE) plot of 32 PCa samples displaying the distribution of cells in different samples in a two-dimensional space. Each point represents a cell, and the color represents different samples. **(B)** Distribution of 15 clusters in the t-SNE plot of PCa samples. Each color represents a cluster of cells, which are clustered on the basis of the gene expression patterns of different cells. **(C)** Distribution of 11 cell types in the t-SNE plot of PCa samples. **(D)** Stacked violin plot showing the annotation marker for the 10 cell types. Each violin chart represents a gene, with the vertical axis representing the gene expression level and the horizontal width representing the distribution of cell numbers at that expression level. **(E)** Heatmap showing the top 5 markers among all cell types. The horizontal axis represents genes, the vertical axis represents cell types, and the colors indicate gene expression levels (from low to high). **(F)** Stacked bar plot showing the cell proportions of 32 samples. Each bar represents a sample, and different colors represent different cell types.

The results of bulk RNA sequencing revealed that the abundance of infiltrating neutrophils in the high-risk group was greater than that in the low-risk group; thus, we explored the specific composition of the TIME of PCa in depth. To further examine the cellular complexities of the PCa TIME, we performed a comprehensive single-cell RNA sequencing analysis. We collected and dissected the GSE181294 dataset (https://www.ncbi.nlm.gov/geo/query/acc.cgi?acc=GSE181294) from a 10X single-cell sequence consisting of 32 samples, including 14 normal tissue samples and 18 PCa tissue samples (**Figure 4A**). We identified 123962 cells following standard quality control and cell filtering, including 62715 cells from tumor tissues and 61247 cells from normal tissues. Fifteen clusters were retained for unsupervised clustering (**Figure 4B**) and were annotated into 11 cell types by representative markers (**Figures 4C–E**): luminal epithelial cells (KLK3, MSMB, and KLK4), basal epithelial cells (KRT19, KRT15, and ELF3), T and NK cells (CD3D, CD2, CCL5, and NKG7), B cells (MS4A1 and CD79A), plasma cells (CD79A and MZB1), macrophages (C1QA, CD68, and CST3), mast cells (CPA3, TPSAB1, and KIT), neutrophils (S100A8, S100A9, and EREG), endothelial cells (IFI27, RAMP2, and SPARCL1), fibroblasts (COL1A2, APOD, DCN, and LUM), and smooth muscle cells (ACTA2, MYL9, TAGLN, and RGS5). Notably, we found a significantly greater proportion of cells in the neutrophil lineage in tumor samples than in normal tissue samples, indicating that neutrophils may be involved in the progression of PCa; this is consistent with our results described above (**Figure 4F**).

### 3.10 Characterization of the heterogeneity of neutrophils in PCa

**Figure 5:**
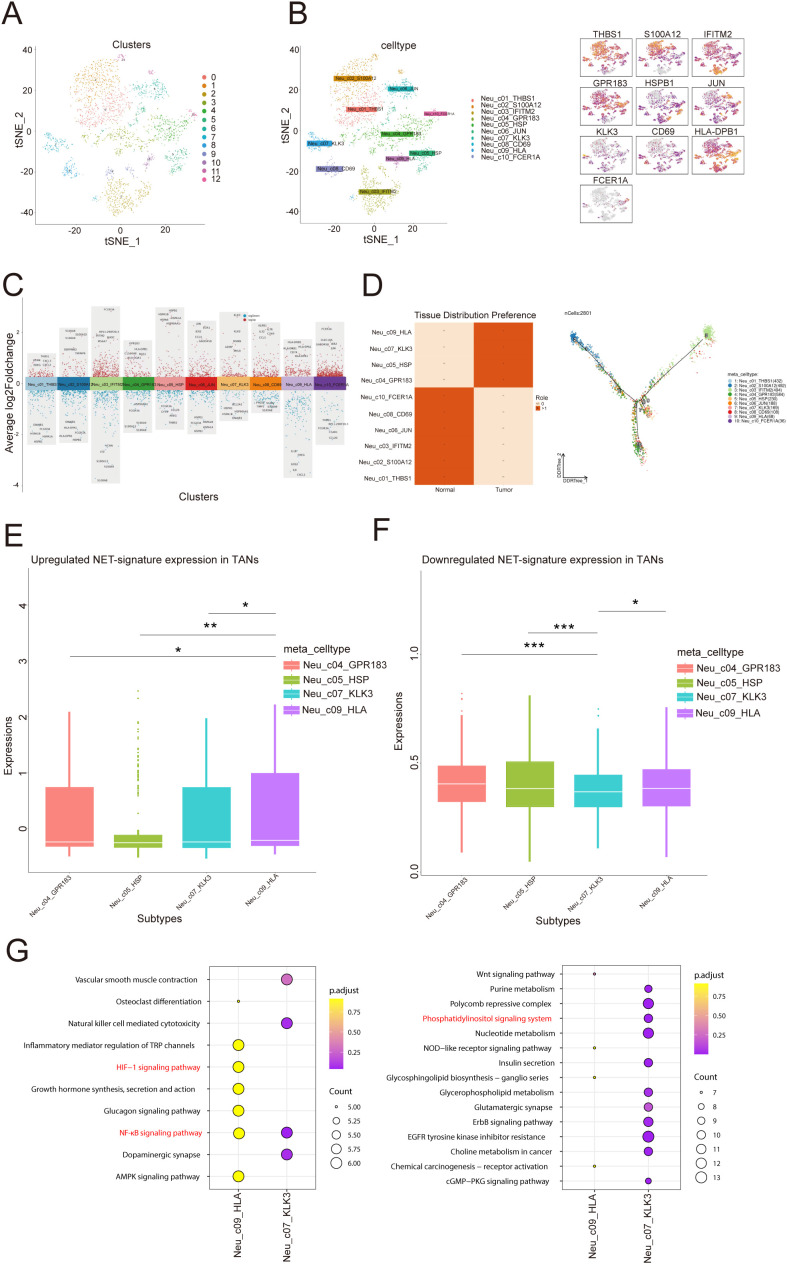
Single-cell transcriptome profiling of neutrophil subtypes. **(A)** Distribution of 12 clusters in the t-SNE plot of neutrophil subtypes. **(B)** Distribution of 10 cell types in the t-SNE plot of neutrophil subtypes and marker gene expression. **(C)** Manhattan plot of highly variable genes of each neutrophil subtype. The change in the average log2 expression value is displayed. The figure shows the expression of specific genes in different cell clusters. **(D)** Heatmap showing the Roe value among all cell types. The figure on the left shows the distribution preferences of different cells in “normal” and “tumor” tissues, with dark squares indicating stronger distribution preferences. The figure on the right shows the topological structure of the cell distribution, with color codes representing different cell types. **(E, F)** The diagram on the left shows that in tumor associated neutrophils (TANs), the upregulation of NET (Neutral Extracellular Traps) marker gene expression. The right figure shows the downregulation of NET marker gene expression in TANs. The differences between different comparison groups are represented by asterisks for statistical significance (*p<0.05, **p<0.01, ***p<0.001). **(G)** Enrichment analysis in two subtypes: Each point represents a gene pathway, the size of the point represents the number of genes, and the color represents the adjusted p-value.

To further explore the biological characteristics of neutrophils in the TIME of PCa, we conducted unsupervised reclustering on neutrophils, obtaining 2801 cells and dividing them into 10 subclusters (**Figures 5A–C**). Thus, we calculated the Roe value of each subtype to distinguish normal-associated neutrophils (NANs) from tumor-associated neutrophils (TANs). These results revealed that Neu_c04_GPR183, Neu_c05_HSP, Neu_c07_KLK3 and Neu_c09_HLA tended to be distributed in tumor tissues, suggesting that they may be TANs, whereas Neu_c01_THBS1, Neu_c02_S100A12, Neu_c03_IFITM2, Neu_c06_JUN, Neu_c08_CD69 and Neu_c10_FCER1A tended to be distributed in normal tissues, suggesting that they may be NANs (**Figure 5D**). Next, we potentially localized 6 risk model genes within 4 TAN subclusters and revealed that Neu_c09_HLA had the highest PSMA1 expression (**Figure 5E**). We constructed a signature of the remaining 5 genes with low expression, and the results revealed that in Neu_c07_KLK3, the expression of the signature was the lowest (**Figure 5F**), suggesting that the biological functions of these two subclusters may be related to the NET prognostic model. Interestingly, Reactome pathway analysis revealed that PSMA1 may be involved in HIF-1α oxygen-dependent proline hydroxylation and NF-κB signaling activation (STRING: functional protein association networks: string-db.org), which was consistent with the involvement of Neu_c09_HLA in the regulation of the HIF-1α signaling pathway (**Figure 5G**); these results suggested that Neu_c09_HLA may regulate hypoxia in the TIME of PCa through PSMA1. Coincidentally, the KEGG pathway analysis revealed that the remaining 5 genes were involved in the phosphatidylinositol signaling system (STRING: functional protein association networks: string-db.org), which was consistent with the regulatory role of Neu_c07_KLK3 in the phosphatidylinositol signaling system (**Figure 5G**). Overall, we analyzed the potential distribution of the 6 genes in TANs and revealed their prospective biological functions, providing evidence for the exploration of molecular targets.

### 3.11 PSMA1 drives neutrophils toward a protumor phenotype

**Figure 6:**
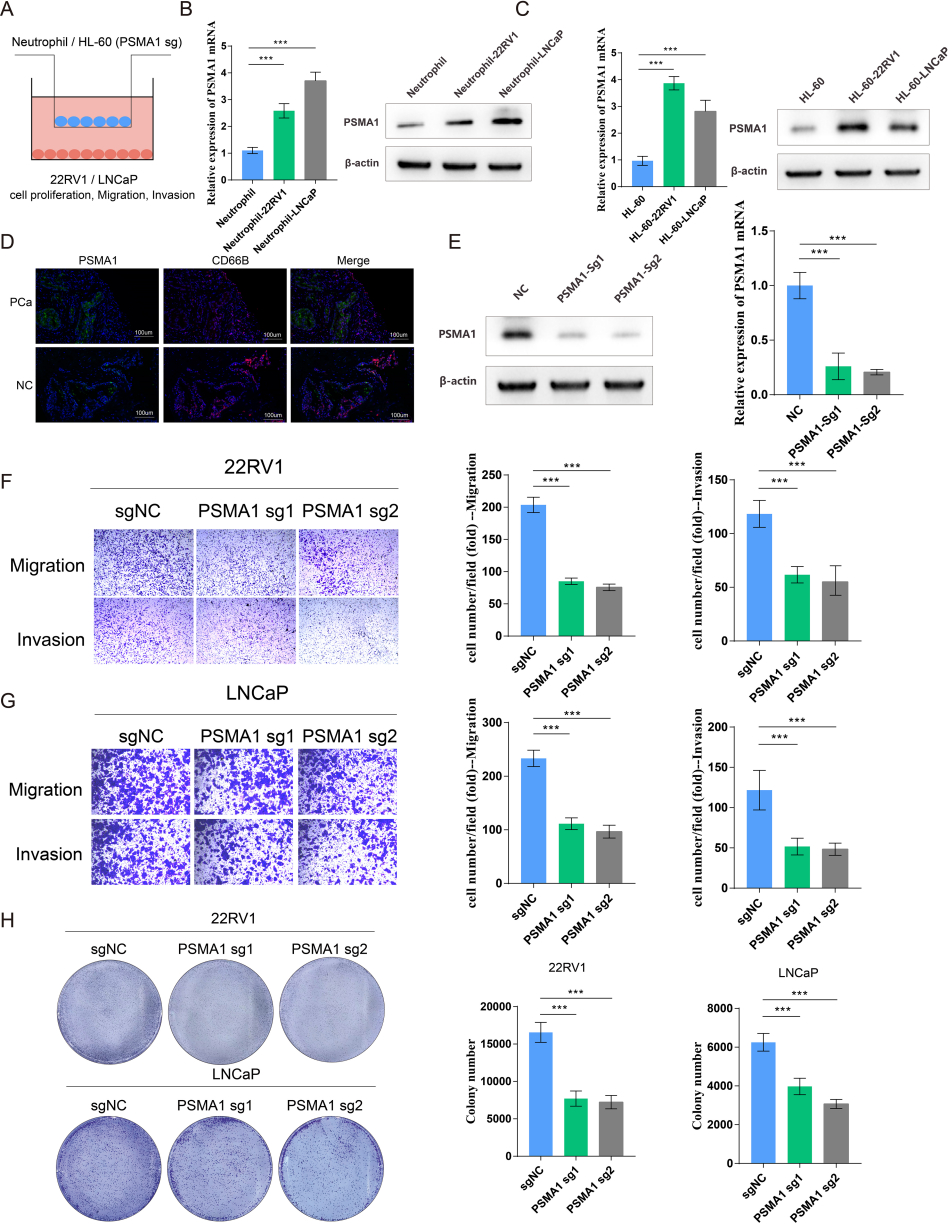
PSMA1 drives neutrophils toward a protumor phenotype. **(A)** Schematic illustration of the HL60 cell/neutrophil-PCa cell coculture system. **(B)** Neutrophils isolated from the peripheral blood of healthy donors were cocultured with 22RV1 or LNCaP cells, and PSMA1 expression was measured by qPCR and WB. **(C)** HL-60 cells were cocultured with 22RV1 and LNCaP cells, and PSMA1 expression was measured by qPCR and WB. **(D)** Immunofluorescence image showing the colocalization of PSMA1 and neutrophils (CD66B) in PCa cancer tissues and normal prostate tissues. **(E)** The knockdown efficacy of PSMA1 was determined by Western blotting and qPCR. **(F, G)** 22RV1 and LNCaP cells were cocultured with control or PSMA1-knockdown HL-60 cells for 2 days, and their migration and invasion capacities were assessed by Transwell assays. The numbers of migrated cells are presented as the means with SDs. **(H)** Colony formation assay, in which control and PSMA1-knockdown HL-60 cells were cultured in the upper chambers, and 22RV1 cells/LNCaP cells were cultured in the lower chambers. The numbers of colonies are presented as the means with SDs. *P<0.05; **P<0.01; ***P<0.001.

**Figure 7:**
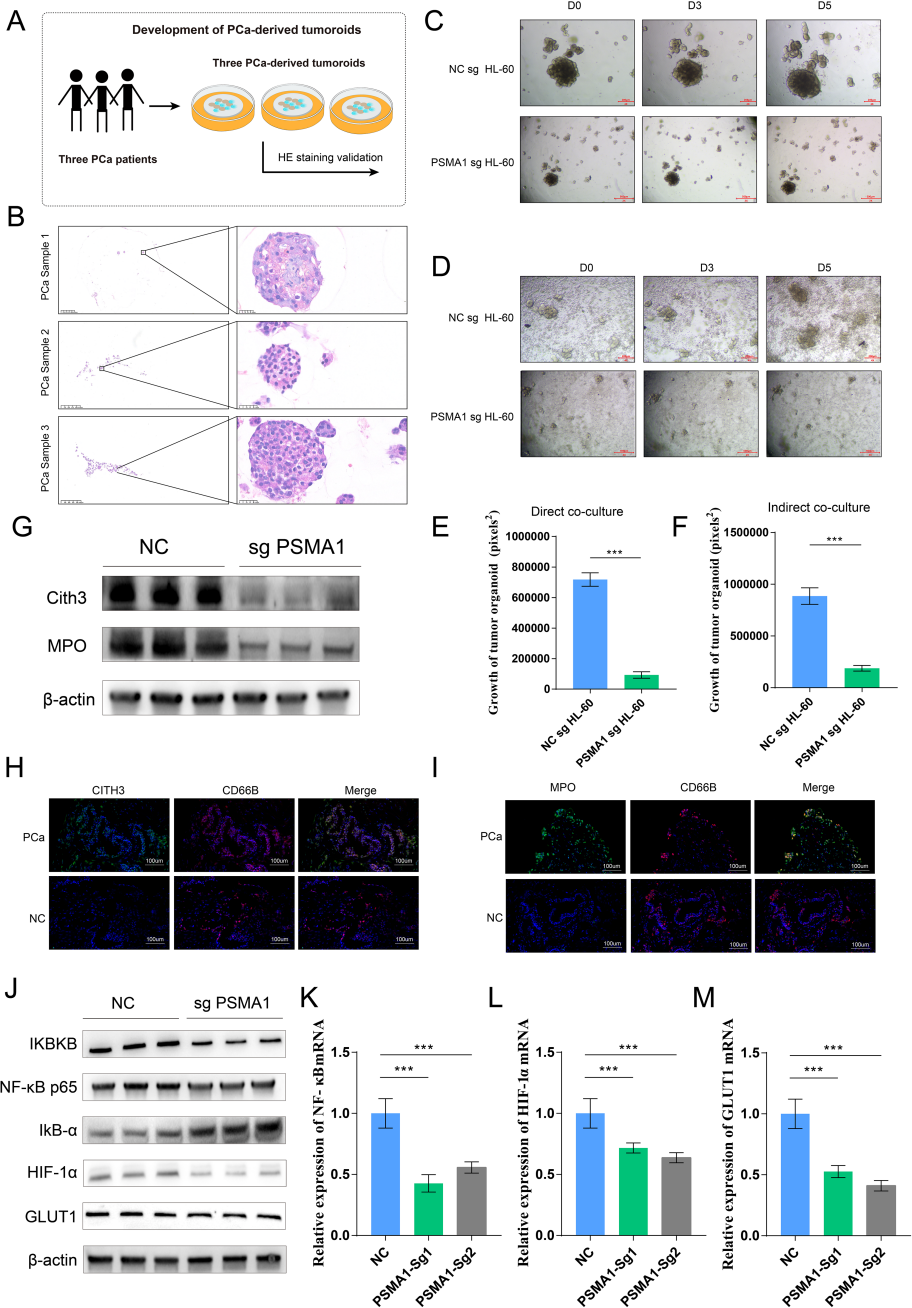
PSMA1-knockdown HL-60 cells inhibited PCa organoid growth and pathway activation. **(A)** Schematic diagram of the establishment of 3 tumor organoids derived from 3 prostate cancer patients. **(B)** HE staining verification of the tissue origin of the constructed tumor organoids. **(C–F)** The conditioned media of NCsg HL-60 cells and PSAM1 sg HL-60 cells were collected and cocultured with organoids, and changes in organoid size were detected. NCsg HL-60 cells and PSAM1 sg HL-60 cells were directly cocultured with organoids, and changes in organoid size were detected. **(G)** Western blotting analysis of the expression of NET markers (MPO and CITH3) in HL-60 cells and PSMA1-knockdown HL-60 cells. **(H, I)** Immunofluorescence image showing the colocalization of NET markers (MPO and CITH3) and neutrophils (CD66B) in PCa cancer tissues and normal prostate tissues. **(J–M)** PSMA1 affects the NF–κB signaling pathway and the activation of hypoxia- and glycolysis-related pathways, as demonstrated by Western blotting and qPCR. *P<0.05; **P<0.01; ***P<0.001.

Considering that tumor-associated neutrophils (TANs) may influence the tumor microenvironment by upregulating PSMA1 to induce hypoxia, we further focused on the role of PSMA1 in neutrophils. We cocultured neutrophils that were isolated from healthy human peripheral blood and HL-60 cells with PCa cells (22RV1 and LNCaP) and observed increases in both PSMA1 mRNA and protein expression (**Figures 6A–C**). These findings demonstrated that PSMA1 levels are elevated in neutrophils and HL-60 cells after they are educated by the PCa TME. In addition, we also demonstrated that the colocalization of PSMA1 and neutrophils (marker CD66B) in PCa tissues was significantly greater than that in normal prostate tissue according to immunofluorescence (**Figure 6D**). On this basis, we established PSMA1-knockdown HL-60 cells and cocultured them with PCa cell lines (**Figure 6E**). We found that the migration, proliferation, and invasion of both PCa lines were reduced, suggesting that low expression of PSMA1 can effectively ameliorate the malignant phenotype of cancer cells (**Figures 6F–H**). Therefore, we successfully established three human-derived PCa organoids and used HE staining to verify the tissue origin of the organoids (**Figures 7A, B**).

On the basis of these findings, we used two methods to demonstrate the effect of PSMA1 in HL-60 cells on organoid growth: ① conditioned media from the NCsg HL-60 cells and PSAM1 sg HL-60 cells were separately collected and cocultured with the organoids, and ② NCsg HL-60 cells and PSAM1 sg HL-60 cells were directly cocultured with the organoids (**Figures 7C, D**). The results revealed that PSAM1 sg HL-60 suppressed the growth of the organoids, further demonstrating the role of PSMA1^+^ TANs in the growth of PCa (**Figures 7E, F**). Furthermore, considering that PSMA1 is a biomarker of NETs, we found that when PSMA1 expression was reduced, the expression of NET markers (Cith3 and MPO) was also decreased (**Figure 7G**); furthermore, immunofluorescence demonstrated that the rate of colocalization between NET markers (Cith3 and MPO) and neutrophils in PCa tissues was greater than that in normal prostate tissue (**Figures 7H, I**), suggesting that PSMA1 can effectively promote the synthesis and release of NETs. Finally, KEGG pathway analysis indicated that TANs activated the NF-κB signaling pathway. Therefore, we hypothesized that TANs upregulate PSMA1, activate the NF-κB signaling pathway, and subsequently regulate the HIF-1α signaling pathway to promote PCa progression. We then demonstrated that the knockdown of PSMA1 inhibited the activation of the NF-κB signaling pathway, as well as the HIF-1α signaling pathway, by WB and qPCR, suggesting that neutrophils influence TME progression through the PSMA1-NF-κB-HIF-1α axis (**Figures 7J–M**). In summary, PSMA1 may be a potential therapeutic target for improving the effects of antitumor therapy.

## 4 Discussion

Prostate cancer is a common and increasingly prevalent malignant tumor in men (18). Despite its prevalence, early diagnosis remains challenging (19). Current prognosis assessments rely on clinical criteria such as serum PSA levels, staging, and histopathology, which fail to accurately predict biochemical recurrence (BCR) and treatment outcomes (20, 21). These findings highlight the critical need for new molecular prognostic markers to better predict risk and treatment response. Neutrophils, which are abundant in peripheral blood, significantly influence inflammatory responses and tumor activity (22). Neutrophil extracellular traps (NETs), which represent a defense mechanism of neutrophils, not only possess antibacterial properties but also promote tumor growth, invasion, metastasis, and immune evasion by altering the tumor microenvironment (23). Current research increasingly suggests that the progression of PCa is closely related to the abundance of tumor-infiltrating neutrophils and NETs (24, 25). In addition, studies have shown that NET-related gene models can accurately predict tumor prognosis and assess immunotherapy response, enhancing patient-specific treatment strategies (26). Given the critical role of neutrophils in the immune microenvironment and prognosis prediction, we aimed to identify NET genes associated with the prognosis of PCa patients.

In this study, we first conducted a retrospective cohort study that revealed a strong association between increased neutrophil counts and more severe clinical complications in PCa patients. In a study of gastric cancer, it was shown that abdominal infection complications (AICs) after gastrectomy can stimulate neutrophils to release NETs, thereby promoting tumor progression and metastasis (27). Considering the close relationship between NETs and neutrophils, we further constructed NET-related models for predicting the prognosis of PCa. We utilized the TCGA dataset to analyze 16045 genes with abnormal expression in PCa. We then intersected these genes with NET-related genes via KEGG, GO, GSEAMSigDB, paper, and Gene Card analyses and found that these NET-DEGs were related to the regulation of the inflammatory response through GO and KEGG analyses. We subsequently identified 12 prognostic NET-DEGs (AGER, ALDOA, FCGR2B, FTH1, HDAC7, HDAC10, ITGA2B, NCF1, NCF4, PLCB2, PLCG1, and PSMA1) through univariate Cox regression analysis. Among the six machine learning algorithms, we selected LASSO on the basis of the best C-index results to construct a prognostic model with 6 genes. The model had a high AUC and good surface predictive ability for prognosis. This prognostic model also served as an independent prognostic indicator for PCa patients according to multivariate regression analysis. Notably, nomograms have been used as effective and reliable clinical tools for evaluating the survival of cancer patients. Therefore, we developed nomograms that involved risk scores and multiple clinical variables (PSA, Gleason score, clinical N stage, and age) to improve the prediction of PCa patient prognosis. Our calibration chart shows that the actual and predicted 1-year, 3-year, and 5-year survival rates based on the column chart are similar. We used different methods to evaluate the differences in immune cell infiltration between high-risk and low-risk PCa patients. We found that high-risk PCa patients had greater infiltration of immune cells, including neutrophils, than low-risk patients. Finally, we predicted new therapeutic drugs that could contribute to the treatment of PCa in different risk groups. The high-risk group was generally more sensitive to these drugs.

In our study, on the basis of scRNA-seq data, we analyzed 32 single-cell samples (16 normal prostate tissue samples *vs*. 16 PCa tissue samples) and identified 10 subclusters, including 4 tumor-associated neutrophil (TAN) subclusters and 6 normal-associated neutrophil (NAN) subclusters. Furthermore, we performed subcluster localization of the risk model with 6 genes in 2 TAN subclusters (Neu_c09_HLA and Neu_c07_KLK3), and the results revealed that these two subclusters may promote tumor progression through dysregulation of hypoxia and the phosphatidylinositol signaling system. Recent studies have shown that neutrophils in the dcTRAIL-R1+ state are located mainly in the glycolytic and hypoxic niches of the tumor core in various cancers (28). In addition, as an important part of the phospholipid signaling system, phospholipid 3-kinase plays a crucial role in various cellular processes and is abnormally activated in cancer, promoting tumor occurrence and progression (29). These findings are consistent with our research.

In cancer, tumor-associated neutrophils can perform dual functions. TANs can promote tumor inflammation by driving angiogenesis, extracellular matrix remodeling, metastasis, and immune suppression. In contrast, neutrophils can mediate antitumor responses by directly killing tumor cells and participating in the cell network that mediates antitumor resistance (30). According to the results of our retrospective cohort study, we hypothesize that neutrophils in PCa primarily mediate a pro-tumor effect. Most NET-DEGs in the TME are associated with prognosis in PCa patients. AGER is overexpressed in cervical cancer cell lines and can promote their proliferation and migration (31). Knockdown of ALDOA in liver cancer inhibits cell growth under hypoxic conditions, leading to delayed tumor growth and inhibition of migration (32). FTH1 is downregulated in PCa tissues and is associated with survival in PCa patients. High expression of FTH1 is associated with a relatively high survival rate (33). Research has shown that the absence of HDAC7 inhibits cell cycle development and reactivates cell aging in a c-Myc-dependent manner, inhibiting the proliferation of human cancer cells, indicating that HDAC7 plays an important role in tumor proliferation (34). Prostate-specific membrane antigen (PSMA1) is overexpressed in the vast majority of PCa cells, and PSMA-guided surgery is currently used mainly for recurrent PCa (35). These data suggest that these 6 prognostic NET-DEGs may affect the progression and prognosis of PCa by influencing the regulation of the immune response. We believe that the potential mechanism by which NET-DEGs affect tumor occurrence and progression deserves further exploration on the basis of our analysis results.

The results of our functional analysis indicate that NET-DEGs are involved mainly in immune-related processes. The tumor microenvironment (TME) is composed of tumor cells and their surrounding stromal cells, which play crucial roles in the occurrence and development of tumors and support the proliferation of tumor cells (36). In the TME, most immune cells exhibit a “double-sided” pattern. They not only can resist tumors but also may promote tumor growth (37). The presence of these immune cells can help suppress cancer cells or regulate their metastasis, invasion, spread, and sensitivity to chemotherapy drugs. The number of immunosuppressive neutrophils is increased in PCa (38), and they support tumor development by inhibiting T cell responses, promoting angiogenesis, treating resistance, and preventing aging (30, 39, 40). In addition, tumor recurrence after treatment is associated with the re-expansion of immunosuppressive neutrophils (41). Therefore, neutrophil infiltration in high-risk PCa patients may be associated with poor prognosis and BCR, and it is closely related to immunotherapy and chemotherapy. The therapeutic effects of cyclophosphamide, 5-fluorouracil, and cisplatin on advanced PCa have been validated in clinical trials (42–44). Studies have shown that the combination of oxaliplatin and 5-fluorouracil (19%) has greater anti-prostate tumor activity than oxaliplatin alone (14%) (45). A high expression level of PLK1 is associated with adverse clinical outcomes. BI2536, which is an effective and selective ATP competitive PLK1 inhibitor, combines low-dose BI2536 with nocodazole (which synchronizes cells in the G2/M phase) to limit side effects and synergistically reduce cell growth and survival in advanced PCa cells (46). These clinical studies demonstrate the basis of our predictions of chemotherapeutics and provide new ideas for future personalized treatment plans. However, the specific mechanisms underlying the differences in drug sensitivity generated by model-based grouping remain unknown, and further exploration should be conducted in the future.

Through a combination of bioinformatics analysis and experiments, we demonstrated that neutrophils regulate the tumor microenvironment via the PSMA1-NF-κB-HIF-1α signaling axis, promoting the malignant progression of tumor cells and thus strongly supporting tumor growth. Studies have shown that PSMA1 can regulate the NF-κB signaling pathway, increase oxidative stress, and thereby drive tumor progression (47). Another study revealed that PSMA1 promotes gastric cancer (GC) progression and proliferation by mediating deubiquitinase activity, suggesting that PSMA1 is a potential therapeutic target for GC (48), which further supports our findings that PSMA1 facilitates prostate cancer progression. Notably, we also established a prostate cancer organoid model to further demonstrate the effect of PSMA1 in neutrophils on PCa progression. Neutrophil extracellular traps (NETs), which are DNA–protein complexes that are released by neutrophils, initially function as antipathogen defense mechanisms. However, in the tumor microenvironment, NETs significantly contribute to immune suppression by inhibiting T cell function, promoting regulatory T cell generation, and suppressing immune cell infiltration, thereby supporting tumor progression and immune escape (49–51). Our study also revealed that reduced PSMA1 levels lead to decreased expression of NET markers in HL60 cells, suggesting that inhibiting PSMA1 may suppress NET formation and improve the immunosuppressive properties of the tumor microenvironment.

However, our study has several limitations. Our retrospective analysis introduced several biases, including selection bias and information bias. While the risk model that was developed uses statistical and machine learning analyses, the generalizability of the model may be limited if the training data do not adequately represent the broader population of PCa patients. In addition, we evaluated only the risk feature prediction of prognosis on the basis of the diagnostic NET-DEGs. Therefore, our next goal is to conduct a more comprehensive study with the aim of elucidating the mechanisms underlying this feature.

## 5 Conclusions

Our study advances the understanding of PCa prognosis by integrating clinical data, machine learning, and bioinformatics analyses. We identified elevated neutrophil levels in PCa patients and developed a 6-gene prognostic model to stratify patients into high- and low-risk groups. Single-cell sequencing revealed that tumor-associated neutrophils (TANs) exacerbate the tumor microenvironment (TME) by upregulating PSMA1, activating the NF-κB-HIF-1α axis, and promoting NET release, which drives malignant progression. A novel NET-related signature is proposed, offering new insights into PCa prognosis and potential therapeutic targets and suggesting a promising direction for enhancing prognostic predictions and personalized treatment strategies; further validation and clinical integration of this signature are warranted to improve patient management and outcomes.

## Data Availability

The datasets presented in this study can be found in online repositories. The names of the repository/repositories and accession number(s) can be found below: https://www.ncbi.nlm.nih.gov/geo/, GSE181294 https://www.ncbi.nlm.nih.gov/geo/, GSE210343.
