# Supplementary material for: Tumor associated neutrophils promote prostate cancer progression by mediating neutrophil trap secretion through PSMA1- NF-κB-HIF-1α signaling axis
Source: Front Immunol. 2025 Aug 18;16:1467357. doi: 10.3389/fimmu.2025.1467357 (PMC12399615; doi:10.3389/fimmu.2025.1467357)
Supplement: Supplementary file 1 [file DataSheet1.pdf]

Supplementary information

Single-cell sequencing combined transcriptomics based on machine learning algorithm to explore the therapeutic and prognostic value of neutrophil extracellular traps in prostate cancer

Table S1. Demographic, clinical and pathological information of the donors for qPCR

ID	Sex	Age	AJCC stage
PCa1	M	65	IIA
PCa2	M	61	IIA
PCa3	M	65	IIB
PCa4	M	59	IIB
PCa5	M	66	IIB
PCa6	M	79	IIB
PCa7	M	68	IIA
prostatic hyperplasia1	M	76	
prostatic hyperplasia2	M	76	
prostatic hyperplasia3	M	65	
prostatic hyperplasia4	M	69	
prostatic hyperplasia5	M	59	

Table S1. qPCR specific primer sequences

RAGEF	TCCAGGATGAGGGGATTTTC
RAGER	GGGAACACCAGCCGTGAG
ALDOAF	GTGTTGTGGGCATCAAGGTAG
ALDOAR	GGCGAAGTCAGCTCCGTC
FCGR2BF	CTTACCTGTCCTTGCCACTGAG
FCGR2BR	AACAGGAGCCAGGAATAGCAC
FTH1F	AGCTCTACGCCTCCTACGTTTAC
FTH1R	AGTTTCTCAGCATGTTCCCTCTC
HDAC7F	AGGACAAGAGCAAGCGAAGTG
HDAC7R	TGTTGGGATGGACTGTTCTTTC
HDAC10F	ATGGGAAACGCTGACTACGTG
HDAC10R	TGAGTCAAATCCTGCCGAGAC
ITGA2BF	CTCCAGTTGCGGATATTTTCTC
ITGA2BR	CCGTCGAAGTACTCTGGGTTG
NCF1F	GGTTGGTGGTTCTGTCAGATGA
NCF1R	GCATAGTTGGGCTCAGGGTC
NCF4F	TACCTGCCCTCAACGCCTAC
NCF4R	GGGGACACGCTCTTGACTTTC
PLCB2F	ATCCTCCGTGTGGATCCTAAG
hPLCB2R	CAAACTTCCCAAAGCGAGTATC
PLCG1F	ATTTCGCCTGAAAACGCTG
PLCG1R	TTCCGATCCACTGAGTAAAACTG
PSMA1F	TATCTCAATTGCGGGGCTTAC
PSMA1R	CACAGGCAGTGGTCTATCGAATAC

Figure S1

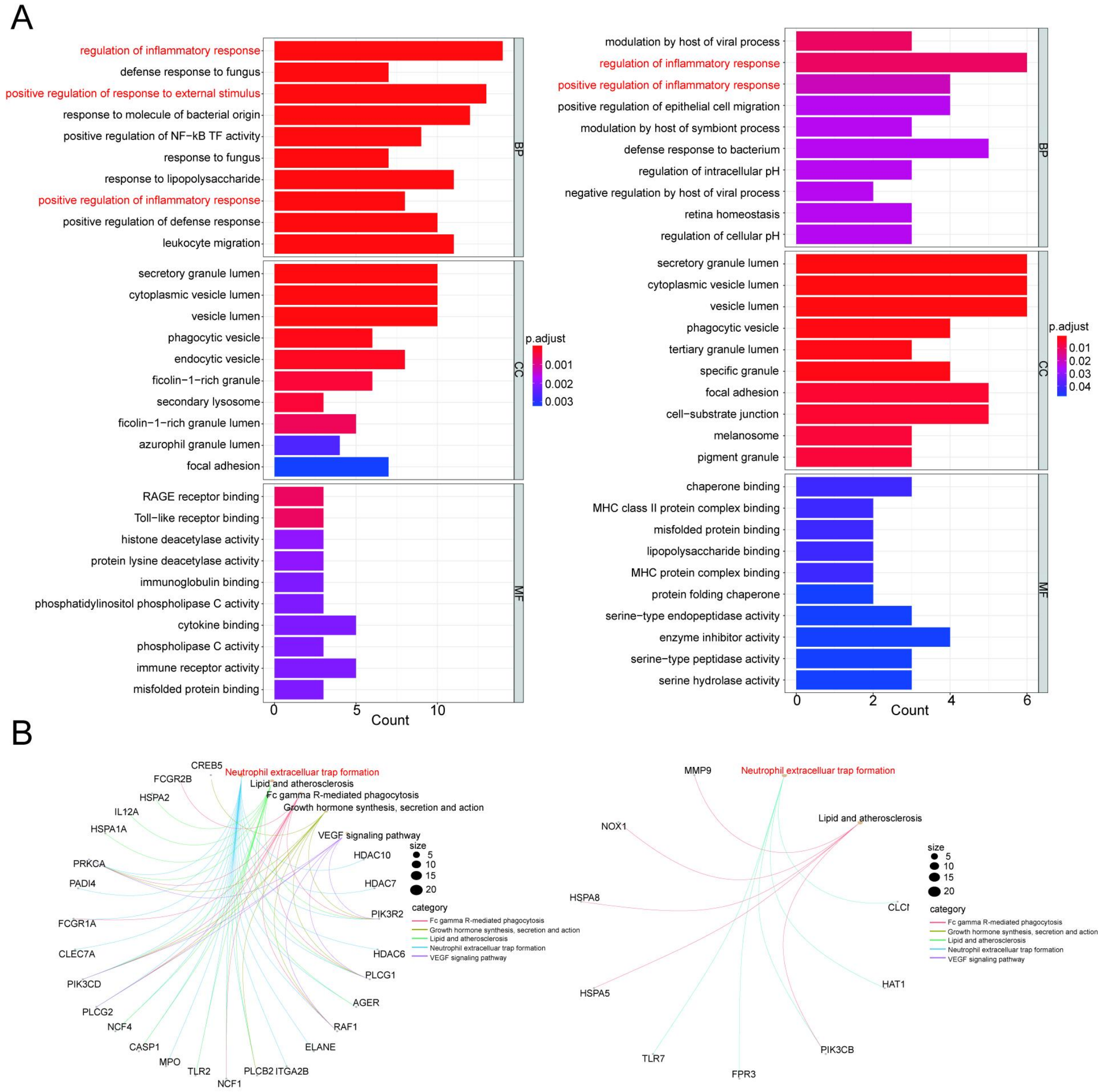

Figure S1: Functional enrichment analysis of NET-DEGs in PCa

A: GO functional analysis of low expression and high expression NET-DEGs in PCa

B: KEGG functional analysis of low expression and high expression NET-DEGs in PCa

Figure S2

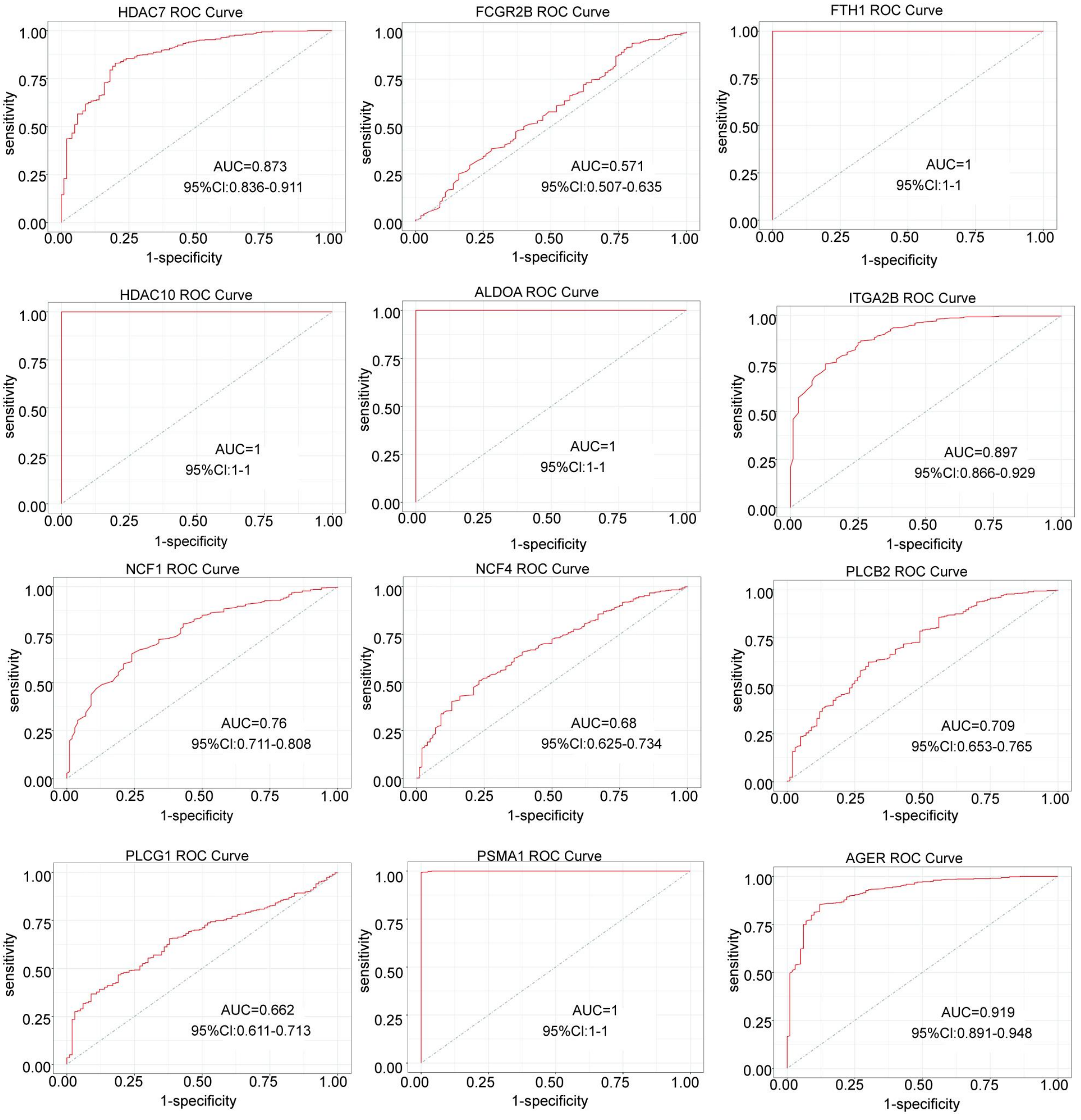

Figure S2: 12 prognostic related NET-DEGs in predicting the ROC curve of BCR: Among them, genes with high predictive performance (AUC close to 1) include: HDAC7, FTH1, HDAC10, ALDOA, ITGA2B, and AGER.

Genes with moderate predictive performance (AUC between 0.5 and 0.8) include: NCF1, NCF4, PLCB2, and PLCG1.

Genes with low predictive performance (AUC close to 0.5 or lower) include FCGR2B and PSMA1.

Figure S3

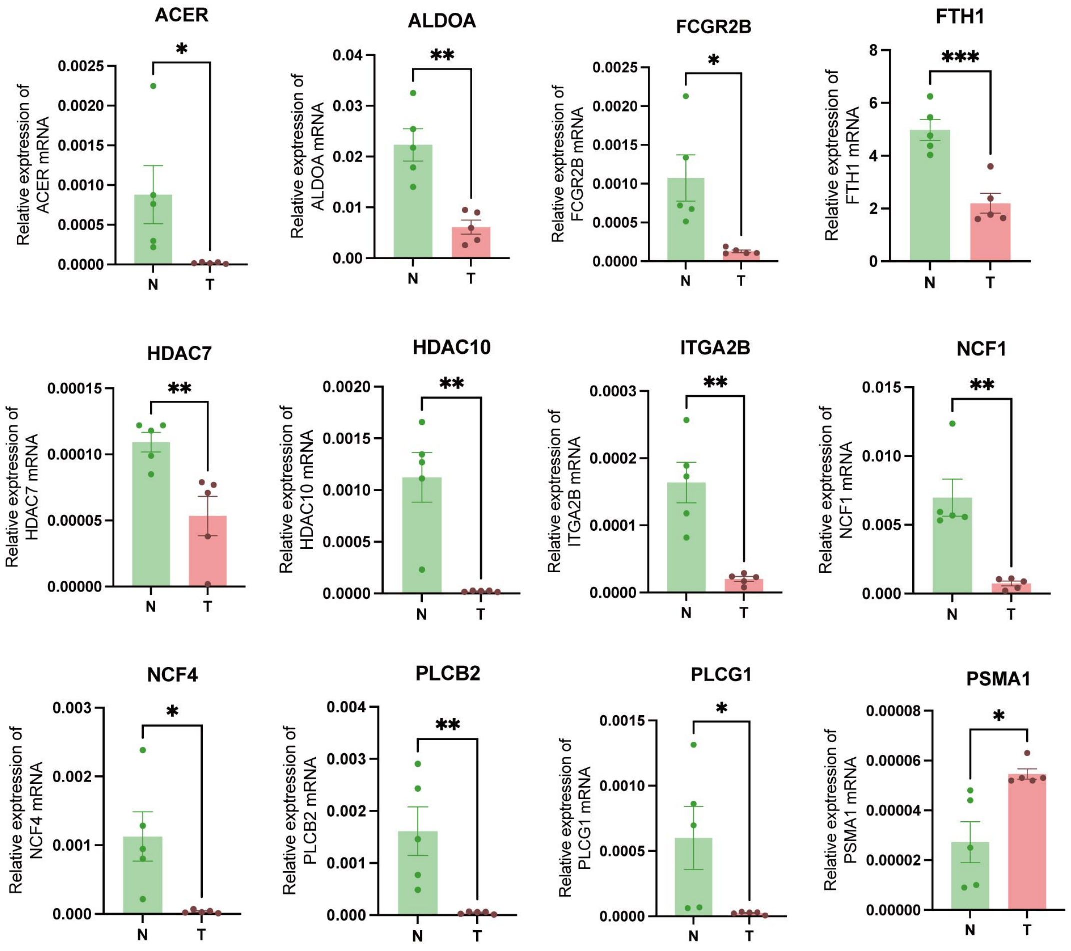

Figure S3: Clinical validation of 12 diagnostic related NET-DEGs expression.

AGER, ALDOA, FCGR2B, FTH1, HDAC7, HDAC10, ITGA2B, NCF1, NCF4, PLCB2, and PLCG1 are significantly downregulated in prostate cancer (normal issue, n=5; PCa issue, n=5). PSMA1 expression is significantly upregulated in prostate cancer. The asterisk represents statistical significance, and the more asterisks there are, the higher the significance (*p<0.05, **p<0.01, ***p<0.001, ****p<0.0001).
